# Oldest Evidence of Toolmaking Hominins in a Grassland-Dominated Ecosystem

**DOI:** 10.1371/journal.pone.0007199

**Published:** 2009-10-21

**Authors:** Thomas W. Plummer, Peter W. Ditchfield, Laura C. Bishop, John D. Kingston, Joseph V. Ferraro, David R. Braun, Fritz Hertel, Richard Potts

**Affiliations:** 1 Department of Anthropology, Queens College and NYCEP, Flushing, New York, United States of America; 2 Research Laboratory for Archaeology and the History of Art, University of Oxford, Oxford, United Kingdom; 3 Research Centre in Evolutionary Anthropology and Palaeoecology, School of Natural Sciences and Psychology, Liverpool John Moores University, Liverpool, United Kingdom; 4 Department of Anthropology, Emory University, Atlanta, Georgia, United States of America; 5 Department of Anthropology, Baylor University, Waco, Texas, United States of America; 6 Department of Archaeology, University of Cape Town, Rondebsoch, South Africa; 7 Department of Biology, California State University, Northridge, Northridge, California, United States of America; 8 Human Origins Program, National Museum of Natural History, Smithsonian Institution, Washington, D. C., United States of America; 9 Paleontology Section, Earth Sciences Department, National Museums of Kenya, Nairobi, Kenya; University at Albany (SUNY), United States of America

## Abstract

**Background:**

Major biological and cultural innovations in late Pliocene hominin evolution are frequently linked to the spread or fluctuating presence of C_4_ grass in African ecosystems. Whereas the deep sea record of global climatic change provides indirect evidence for an increase in C_4_ vegetation with a shift towards a cooler, drier and more variable global climatic regime beginning approximately 3 million years ago (Ma), evidence for grassland-dominated ecosystems in continental Africa and hominin activities within such ecosystems have been lacking.

**Methodology/Principal Findings:**

We report stable isotopic analyses of pedogenic carbonates and ungulate enamel, as well as faunal data from ∼2.0 Ma archeological occurrences at Kanjera South, Kenya. These document repeated hominin activities within a grassland-dominated ecosystem.

**Conclusions/Significance:**

These data demonstrate what hitherto had been speculated based on indirect evidence: that grassland-dominated ecosystems did in fact exist during the Plio-Pleistocene, and that early *Homo* was active in open settings. Comparison with other Oldowan occurrences indicates that by 2.0 Ma hominins, almost certainly of the genus *Homo*, used a broad spectrum of habitats in East Africa, from open grassland to riparian forest. This strongly contrasts with the habitat usage of *Australopithecus*, and may signal an important shift in hominin landscape usage.

## Introduction

**Figure 1:**
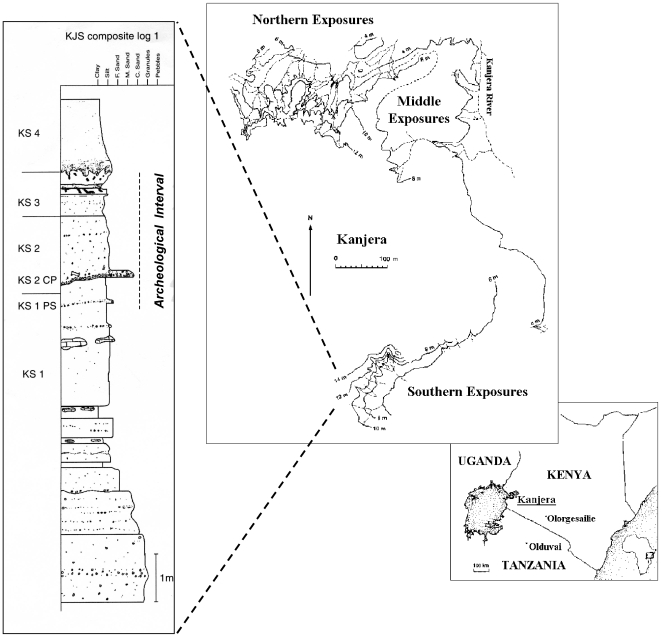
Placement map and stratigraphic diagram showing the location of Kanjera in southwestern Kenya and of the Southern Exposures at Kanjera. The composite stratigraphic log shows the basal three beds of the Southern Member (KS-1 to KS-3) and the base of KS-4. Spatially associated artifacts and fossils are found as diffuse scatters and also in more vertically discrete concentrations from the top of KS-1 through KS-3, with KS-2 providing the bulk of the archeological sample.

The hominin fossil and archeological records of Africa exhibit substantial anatomical and behavioral change during the Plio-Pleistocene (∼1.5–3.0 Ma), including the evolution of *Homo* and *Paranthropus*, the origin of lithic technology and archeological sites, the first evidence of large mammal butchery, lower limb elongation and selection for endurance running, and thermoregulatory adaptations to hot, dry environments [1]. These evolutionary innovations have been linked consistently to novel selective pressures encountered as early hominins foraged increasingly in more open, arid woodland and grassland habitats that were replacing wooded biomes. However, the most finely resolved evidence for environmental change is not from the fossil and archeological sites themselves, but from deep sea core records that indicate drier and more variable conditions in tropical and subtropical Africa [2]–[8]. An increase in arid-adapted vegetation is also reflected by morphological changes across many African large mammal lineages and by the dispersal of the Eurasian grazer *Equus* across Africa ∼2.3 Ma [9], [10]. Although these data suggest that grassland-dominated ecosystems (defined here as having >75% C_4_ plants and a graze-dependent fauna) should be present as one extreme of the continental habitat spectrum, actual documentation of both Pliocene grasslands and of hominin activities in open habitats has until now eluded paleoanthropologists. Here we use faunal and stable isotopic evidence to demonstrate the earliest presence of a grassland-dominated ecosystem, and archeological evidence for hominin activities within this setting from the late Pliocene locality of Kanjera South, Kenya (Fig. 1). At least one species of tool-making hominin, almost certainly of the genus *Homo* [1], was repeatedly using this open setting. In contrast, most other Oldowan occurrences are situated in more wooded settings. These findings indicate that by ∼2.0 Ma tool-making hominins, probably early *Homo*, accessed and used a broad spectrum of East African habitats, from open grassland to riparian forest.

The late Pliocene Oldowan occurrences at Kanjera South are found on the northern margins of the Homa Mountain Carbonatite Complex, Homa Peninsula, southwestern Kenya (Fig. 1). The Homa Peninsula lies within the Nyanza Rift, which presented a depositional low to the north of the site. The lithological sequence at Kanjera South consists of 6 beds of the Southern Member of the Kanjera Formation, from oldest to youngest KS-1 to KS-6 [11], [12]. Only KS-1 to KS-4 is described here since archeological occurrences are known only within this interval, from the top of Bed KS-1 through Bed KS-3.

KS-1 deposition began as a flow of pyroclastic material, possibly as a lahar, from the Homa Mountain complex in the south towards the depocenter in the Nyanza Rift graben. Lower KS-1 shows little internal stratification and no pedogenic development. In contrast, the well-bedded, better sorted and pedogenically modified upper parts of KS-1 represent reworking of the deposits by ephemeral streams running across the fan of the original pyroclastic flows. KS-2 represents a continuation of this environmental setting, with deposition by anastomosing channels flowing with intermittent, diffuse, generally low energy flow regimes and better-developed pedogenesis than KS-1.

KS-3 sees the transition to a wetter depositional environment, as evidenced by soft sediment deformation and the presence of a small channel, though stable land surfaces with pedogenesis continued to be found. KS-4 represents a continuation of this moister trend, with clays being deposited either during the transgression of a lake out of the depocenter to the north or during the formation of a wetland system. The homogeneity of the KS-4 clays favors the former interpretation, and the paleosol layers interbedded in KS-4 indicate intervals of lake regression sufficiently long for pedogenesis to take place.

A combination of biostratigraphy (co-occurrence of the equid *Equus* sp., the suids *Metridiochoerus andrewsi* and *M. modestus*, and the proboscidean *Deinotherium* sp.) as well as magnetostratigraphy (a reversed sequence in Beds KS-1 to KS-4 with the presence of the Olduvai subchron (1.95–1.77 Ma) in Beds KS-5 and KS-6) indicate that the KS-1 to KS-3 archeological occurrences date between ∼2.3 Ma (the dispersal of *Equus* across Africa, first occurrence of *M. modestus*) and 1.95 Ma (the base of the Olduvai subchron) [1], [11]. Given the apparent rapidity of deposition, an age of ∼2 Ma for the archeological occurrences seems most likely.

Except for artifacts and fauna found in thin, discontinuous conglomerate lenses, hominin activity was the primary agent of accumulation of the majority of archeological materials at this site [11], [13]. Discussion here focuses on KS-2 materials from the 169 m^2^ Excavation 1, which has yielded 2190 fossils and over 2471 artifacts with three dimensional coordinates from several levels within the 1.5-m-thick sequence. KS-2 accumulated rapidly and, based on the limited development of pedogenic features, likely represents decades to centuries of deposition.

## Results and Discussion

**Figure 2:**
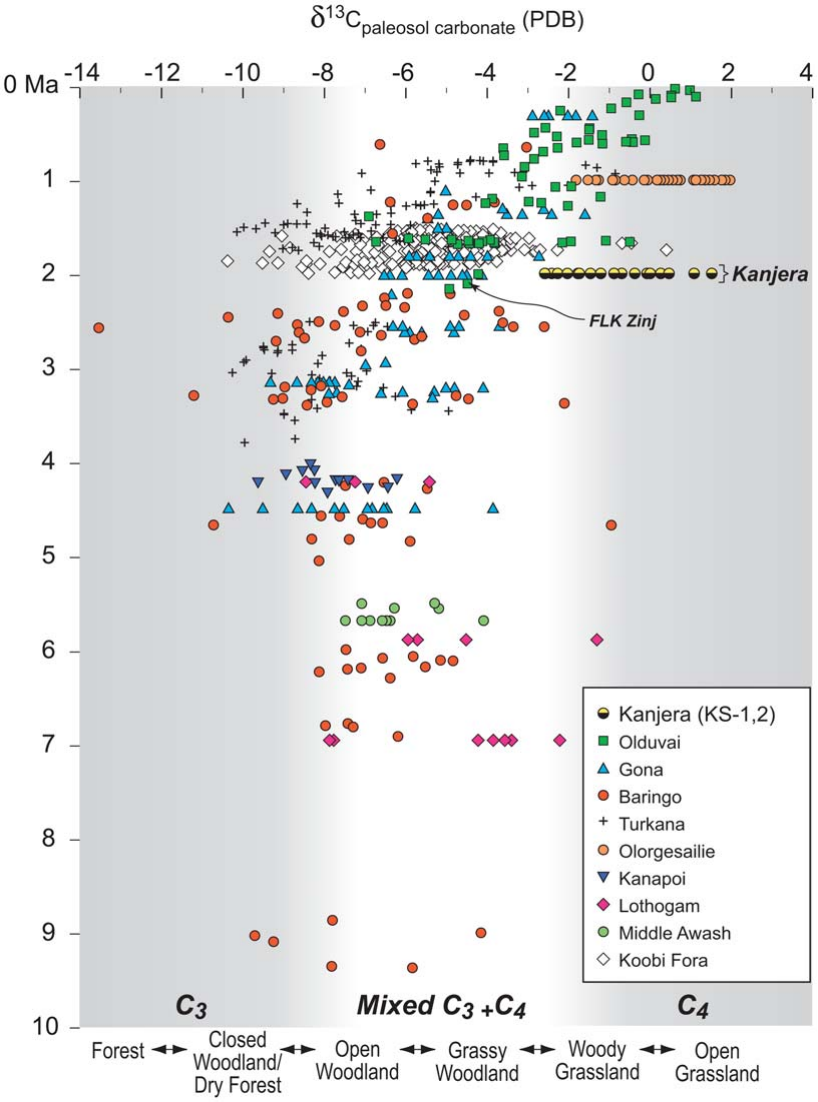
Stable carbon isotopic composition of paleosol carbonates from the late Miocene through Pleistocene of East Africa [7], [11], [30], [33], [51]–[59]. Shaded intervals represent pedogenic nodules forming in C_3_ dominated and C_4_ dominated environments. Paleosol carbonate δ^13^C values of -2 or greater are approximately equivalent to floral communities with 75% or more C_4_ plants [31]. Intermediate values represent a mix of C_3_ and C_4_ vegetation. Plant root systems associated with KS-2 archeological site formation could have extended into the top of KS-1, so paleosol carbonate data from both KS-2 and the top of KS-1 are presented.

Habitats rich in plants using the C_3_ photosynthetic pathway, such as woodland and dry forests, are well-documented between 10 and 2 Ma in East Africa (Fig. 2). Stable isotopic analysis of pedogenic carbonates and occluded paleosol organic matter across an 130 m transect of the Kanjera South locality provide the first clear evidence of a grassland setting (>75% C_4_ vegetation) in this 10 million year sequence (Fig. 2, Tables S1 and S2). Evidence that grassland habitats dominated the regional ecosystem beyond the confines of our excavations is provided by large mammal frequencies, particularly of the family Bovidae, as well as the stable isotopic composition of tooth enamel from a suite of herbivorous mammals. Large mammals often range extensively during the course of a season or year [14], [15] and so can provide a sense of regional vegetation structure [16]. Predicted habitat and dietary preferences of primates, ungulates and proboscideans from Kanjera (Table S3) are based primarily on analogy with extant relatives, degree of hypsodonty, functional analysis of limbs and masticatory morphology, and stable isotopic analyses of South and East African fossil and modern fauna [9], [17]–[27]. Several taxa (crocodile, Phalacrocoracidae [cormorant], hippopotamid) reflect proximity to permanent water, perhaps a lake as suggested by KS-4. The reduncine bovids are indicative of edaphic grasslands and possibly woodland along the lake margin, whereas woodland is suggested by the presence of several tragelaphine bovid fossils, giraffe remains, and a *Cercopithecus* sp. monkey. The suid *M. modestus* may also signal woodland [18]. A *Hippotragus* sp. bovid fossil signals a woodland/grassland ecotone, whereas the antilopine *Antidorcas recki* is best associated with bushland to grassland habitats [9], [22], [24], [25]. The equids, alcelaphine bovids, and *Theropithecus* fossils are indicative of open, grassy environments. In spite of the range of predicted habitat preferences, taxa that preferred open, grassland habitats dominate the fauna (Tables S3 and S4). Seven hundred thirty two of the 886 fossils (82.6%) attributable to zoological family are bovids; of the 143 fossils identifiable to tribe, 132 (92%) are Antilopini or Alcelaphini, which are indicators of open grassland ecosystems in modern settings [9], [27]. The high frequency of equids in KS-2 (11.6%) is similar to the relative abundance of zebras in modern, grassland-dominated ecosystems in East Africa such as the Serengeti, Tanzania [11], [28].

**Figure 3:**
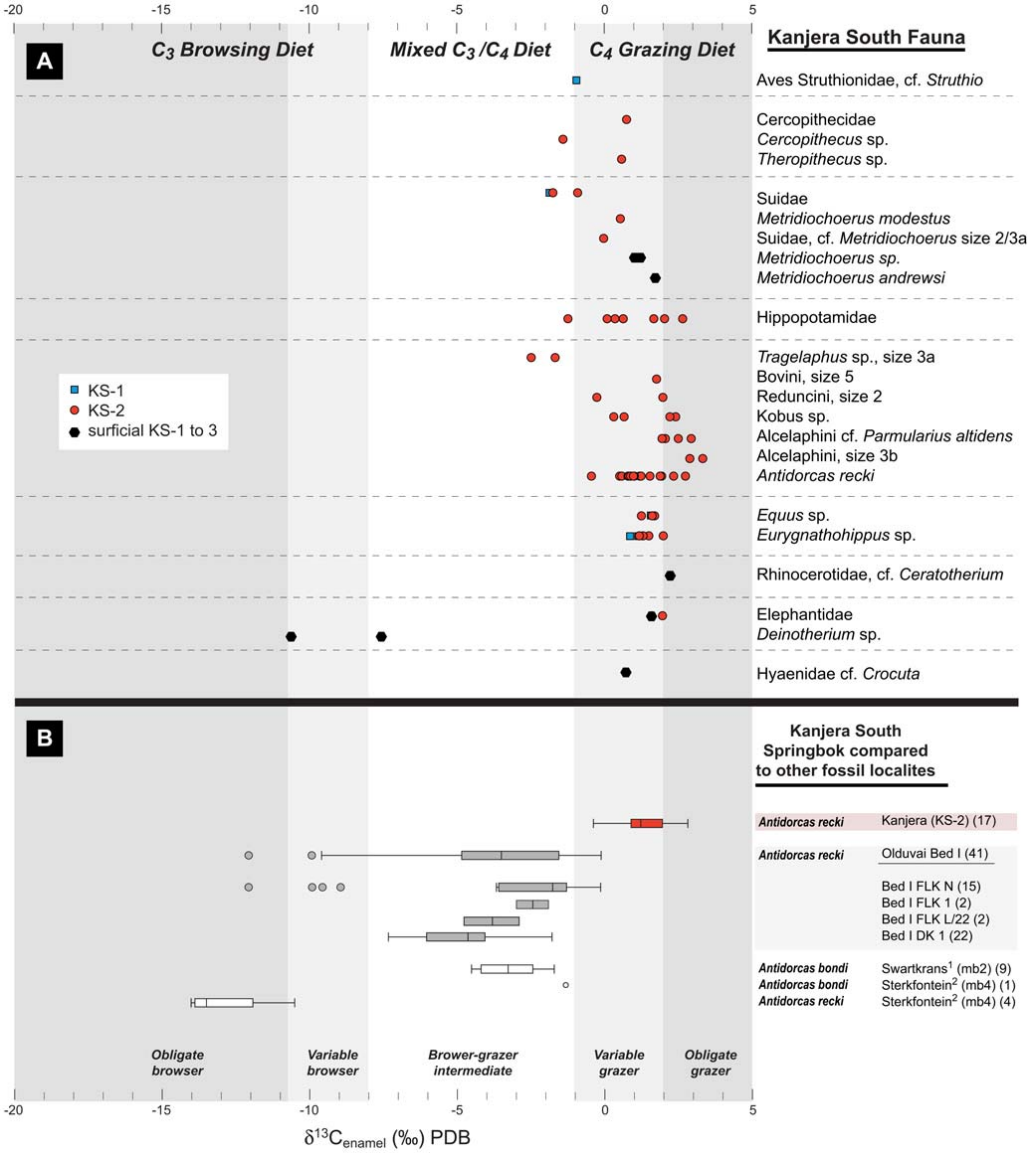
Stable carbon isotopic data of enamel from Kanjera South and other African localities. A. Stable carbon isotopic composition of fossil mammal tooth enamel from KS-2 in Excavation 1. The KS-2 fauna is supplemented by several taxa unique to KS-1 or KS-3, or found on the surface of the KS-1 to KS-3 sequence, to provide a more complete sense of the diet of the mammalian community during the deposition of the archeological levels. The shading reflects the relative importance of C_3_ browse versus C_4_ grass in the diet, with δ^13^C values greater than -1 reflecting a diet with more than 75% C_4_ vegetation. Isotopic dietary classification follows others [23]. A probable ostrich (cf. *Struthio*) eggshell fragment was also analyzed. B. Box and whiskers plots of the stable carbon isotopic composition of modern and fossil gazelles from Kanjera, Bed I Olduvai Gorge, Tanzania, and Sterkfontein and Swartkrans, South Africa [60], [61]. Like many modern antilopines, *Antidorcas recki* was able to switch between browse and graze as necessary [9]. Numbers in parentheses after site name represent number of samples analyzed. Bed I localities are presented in stratigraphic order, from oldest (DK I, ∼1.87 Ma) to youngest (FLK NI, ∼1.78 Ma) [3].

Isotopic analysis of enamel indicates that these taxa uniformly had a large amount of grass in their diets, reflecting the dominance of grass in the vegetation community (Fig. 3a) (Tables S3 and S4). This is true even for taxa that normally have a C_3_-rich (fruit or browse) diet (e.g., tragelaphine bovids and the monkey *Cercopithecus* sp.). One of the two teeth from *Deinotherium*, an obligate browser, has the most positive δ ^13^C value ever documented for this taxon [23], [29]. This indicates *Deinotherium* at least occasionally consumed C_4_ plants. The strong C_4_ signal occurs across the spectrum of animals found at Kanjera South, including non-dispersing taxa such as monkeys, rhinos, tragelaphine bovids, and suids. This confirms that the grassland dietary signal is not simply the result of dry season domination of the residential mammalian community by migratory grazers congregating near a permanent water source.

These data provide the earliest isotopic evidence of an open habitat and a grassland-dominated ecosystem in East Africa. The presence of artifacts and archeological fauna both low and high in the KS-2 sequence and in the underlying KS-1 and overlying KS-3 indicates that hominins repeatedly visited this grass-rich area on the landscape for hundreds or even thousands of years. These data also substantively expand the known range of variation in Oldowan hominin habitat usage. Paleosol carbonate studies from the type locality of the Oldowan Industrial Complex, Olduvai Gorge, Tanzania, suggest that the Bed I and lower Bed II (∼1.7–1.87 Ma) basin margin was frequently well-wooded [30], [31]. Paleosol carbonate isotopic chemistry from the most informative Bed I archeological occurrence, FLK I Level 22 (FLK Zinj), suggests that artifacts and fossils were deposited in a grassy woodland (Fig. 2). Stable isotopic analysis of enamel samples from the extinct gazelle *Antidorcas recki* document an increasing amount of graze and a decreasing amount of browse in their diet through the Bed I sequence (Fig. 3b), consistent with a drying trend noted by other lines of paleontological and geological evidence [24], [28], [32]. Enamel samples of *A. recki* from FLK Zinj suggest a mixed diet of browse and grass, whereas Kanjera *A. recki* was predominantly grazing. *Antidorcas recki* individuals from the relatively arid, upper portion of the Bed I sequence have more negative δ^13^C values than those in the Kanjera KS-2 sample, suggesting that there was a greater proportion of open habitat at Kanjera than at any time during the deposition of Bed I Olduvai. Isotopic data at or in the vicinity of other Oldowan occurrences, including the oldest archeological sites (2.5–2.6 Ma) at Gona, Ethiopia [33] and 1.75–2.0 Ma occurrences at Koobi Fora, Kenya [34] indicate hominin activities in habitat mosaics that on average had 50% C_3_ vegetation (Fig. 2). Pollen data from Gona is concordant with a well-wooded setting for hominin site activities [35]. Vegetation mosaics including substantive woodland components are also suggested for Pliocene and early Pleistocene Oldowan sites in West Turkana, Kenya, and the Shungura Formation, Ethiopia [36], [37]. Finally, Oldowan hominin activities in a riparian forest setting are suggested by paleoenvironmental evidence from the Koobi Fora Formation in the Turkana basin, Kenya, at ∼2.0 Ma [38].

These findings indicate that by ∼2.0 Ma Oldowan hominins had access to and used a broad spectrum of East African habitats, from open grassland to riparian forest. Stone tool manufacture and archeological site formation at this time is most likely attributable to the genus *Homo*. Associations between *H. habilis sensu lato* (here including *H. habilis* and *H. rudolfensis*) and stone tools are known in the geological record by 2.3 Ma [39], [40]. The single definitive stone tool user in the Plio-Pleistocene, *H. erectus* (here including *H. ergaster*), appears in Africa by 1.8 Ma [41]. Overlap in size, cranial morphology, and cranial scaling between *H. habilis* and *H. erectus* suggest a close phylogenetic relationship between the two species [42]–[44], and support the idea that late Pliocene stone tool use was part of the behavioral repertoire of the evolving *Homo* lineage. Brain size expansion and masticatory changes in the *Homo* lineage have plausibly been linked to stone tool-dependent foraging [1].

*Paranthropus*, also known prior to 2.0 Ma, has been argued to have made stone tools based on hand bone morphology, and its stratigraphic association with Oldowan artifacts in eastern and southern Africa [45]. However, the developmental investment in very large jaws and cheek teeth seen in *Paranthropus* would have been unnecessary if a stone tool kit allowing extra-oral processing of food was in use. Moreover, there is no perceptible change in the archeological record after *Paranthropus* goes extinct, as might be expected if two parallel tool traditions, one formed by *Homo*, the other by *Paranthropus*, were in place during the late Pliocene and early Pleistocene [1]. While *Paranthropus* may have used a non-lithic technology [46], it is unlikely to have formed the Oldowan occurrences under consideration here.

The breadth of habitat use inferred for early *Homo* by 2.0 Ma contrasts strongly with that of *Australopithecus*, a precursor to *Homo*, for which heterogeneous environments, all with significant woodland or forest components, are documented consistently [23], [47]. The ∼1.5 Ma *H. erectus* skeleton from Nariokotome, Kenya, signals adaptive shifts in hominin mobility, foraging, and thermoregulation towards the increased use of open, hot, and dry environments [48]–[50]. These shifts are anticipated by the recurrent use of open habitats by early *Homo* at Kanjera. The Kanjera data do not, however, necessarily indicate that early *Homo* used open habitats in preference to wooded ones. Combined evidence from Oldowan sites suggests that early *Homo* was flexible in its habitat use, and that the capacity to extract resources from a range of open and more wooded environments was a vital component of its adaptation.

## Methods

Excavation 1 was carried out within a grid of 169 1m×1m squares, excavated in 5 cm spits following site stratigraphy. Fossil and artifact-bearing horizons were dug with awls and dental picks. A Topcon total station was used for the precise determination of specimen N, E, and Z coordinates and in contour mapping. Object dip and orientation was measured with a Brunton compass. Sediments were dry sieved through 1 mm mesh. Sedimentary, taphonomic, and zooarcheological analyses indicate that the site assemblages formed predominantly through hominin activity [11], [13]. In KS-1, KS-2 PS, and KS-3 there is a clear spatial relationship between the artifacts and fauna. Many objects are outsized clasts relative to grain size; a diverse array of skeletal parts exhibiting a range of hydraulic transport potentials have been recovered; artifact and fossil refits have been made; and both percussion marks and cut marks have been found on bones.

### Isotopic Analysis of Paleosol Carbonates

Pedogenic carbonates used in this analysis exhibited microstructure consistent with in situ formation without subsequent recrystallization. The difference in the δ ^13^C of occluded organic matter and pedogenic carbonate fits theoretical predictions for diagenetically unaltered materials. Diagenetic carbonate cements from the Kanjera Formation have negative δ ^13^C values (Table S1), so that the positive signal reported here is unlikely to have resulted through diagenetic alteration.

Paleosol carbonate samples were washed in double distilled water and dried. The outer layers of the carbonate nodules were removed using a dental burr and discarded. The inner part of each carbonate nodule was crushed in an agate mortar and each sample was split into two aliquots, the first treated with 2% NaOHCl solution at 60°C for 24 hours to remove any organic contamination. The second aliquot was treated with 1M HCl until no reaction was observed and the remaining organic matter was washed to neutrality and freeze-dried. Organic samples were analyzed using flash combustion CF-IRMS. Samples were combusted in a Carlo Erba 1108 sample converter and the evolved gas was analyzed in a Europa Geo 20/20 gas source mass spectrometer at the University of Oxford. Results are reported using the standard delta per mil (‰) notation relative to the VPDB international standard (Table S2). International and in-house standards analyzed along with the organic samples gave standard deviation of ±0.4‰ for carbon.

### Isotopic Analysis of Enamel

Tooth enamel samples were carefully cleaned using an aluminium oxide air abrasive system to remove any adhering sediment and cementum. The outer surface of the enamel was abraded further, removing the outermost portion that was most likely to be diagenetically altered. Samples were then extracted from the cleaned enamel using a 0.5 mm diamond dental burr. Samples were ground and homogenized using an agate mortar. Powdered enamel samples were treated with 2% NaOHCl solution at 60°C for 24 hours to remove any organic contamination. Samples were then washed with double distilled water and treated with 0.1M CH_3_COOH at 25°C for 6 hours under vacuum to remove any secondary carbonate contamination. Samples were rinsed to neutrality and dried. All enamel samples for isotopic analysis were reacted with 100% phosphoric acid at 90°C in a common acid bath system. The evolved CO_2_ was pre-concentrated using a cold finger system and was analyzed at the University of Oxford using a VG Prism gas source isotope ratio mass spectrometer running in dual inlet mode. Results are reported using the standard delta per mil (‰) notation relative to the VPDB international standard. International and in-house standards analyzed along with the enamel samples gave standard deviations of ±0.08‰ for carbon and ±0.12‰ for oxygen.

## Supporting Information

Table S1 Isotopic data from diagenetic sparry, pendant and poikilotopic calcite cements from KS-1 and KS-2, and from samples of carbonatite from the Homa Mountain carbonatite complex.
(0.03 MB DOC)

Table S2 Paleosol Carbonate Isotopic Data.
(0.04 MB DOC)

Table S3 Vertebrate taxon list from KS-2, Excavation 1. Isotopic dietary classification of Kanjera mammalian fossils follows others (23) using the isotopic data presented in Table S4. Obligate grazers and obligate browsers consume an almost exclusive (>95%) C4 or C3 diet, respectively. Variable grazers and variable browsers consume a predominantly (75–95%) C4 or C3 diet, respectively. Brower-grazer intermediate refers to taxa consuming a mix of C4 and C3 vegetation.
(0.04 MB DOC)

Table S4 Stable isotopic composition of fossil eggshell and tooth enamel from Excavation 1.
(0.13 MB DOC)
