# Supplementary material for: Oldest Evidence of Toolmaking Hominins in a Grassland-Dominated Ecosystem
Source: PLoS One. 2009 Oct 21;4(9):e7199. doi: 10.1371/journal.pone.0007199 (PMC2746317; doi:10.1371/journal.pone.0007199)
Supplement: Table S1 — Isotopic data from diagenetic sparry, pendant and poikilotopic calcite cements from KS-1 and KS-2, and from samples of carbonatite from the Homa Mountain carbonatite complex. (0.03 MB DOC) [file pone.0007199.s001.doc]

| **Sample** | **δ^18^O PDB**  **(Craig corrected)** | **δ^13^C PDB**  **(Craig corrected)** |
| --- | --- | --- |
| Kjpwdd1 | -8.93 | -7.60 |
| Kjpwdd2 | -9.54 | -8.40 |
| Kjpwdd3 | -8.92 | -7.40 |
| Kjpwdd4 | -9.32 | -8.45 |
| Kjpwdd5 | -5.66 | -8.30 |
| Kjpwdd6 | -8.86 | -8.20 |
| Kjpwdd7 | -8.47 | -8.80 |
| Kjpwdd8 | -6.74 | -6.30 |
| Kjpwdd9 | -7.30 | -9.50 |
| Kjpwdd10 | -9.57 | -8.10 |
| Kjpwdd11 | -7.41 | -6.20 |
| Homa Mountatin carbonatite samples |  | -4.8 to -8.0 |
