# Supplementary material for: Oldest Evidence of Toolmaking Hominins in a Grassland-Dominated Ecosystem
Source: PLoS One. 2009 Oct 21;4(9):e7199. doi: 10.1371/journal.pone.0007199 (PMC2746317; doi:10.1371/journal.pone.0007199)
Supplement: Table S2 — Paleosol Carbonate Isotopic Data. (0.04 MB DOC) [file pone.0007199.s002.doc]

| **Sample** | **KS bed** | **δ^18^O PDB**  **(Craig corrected)** | **δ ^13^C PDB**  **(Craig corrected)** |
| --- | --- | --- | --- |
| 1481F | 1 | -3.87 | -2.55 |
| 1481G | 1 | -5.20 | 1.11 |
| HP-95-46/1 | 1 | -5.02 | -0.35 |
| HP-95-46/2 | 1 | -9.93 | -1.42 |
| HP-95-46/3 | 1 | -6.02 | 0.04 |
| HP-95-46/4 | 1 | -7.79 | -0.33 |
| KJS-96-17/1 | 1 | -7.69 | -1.70 |
| KJS-96-6b/1 | 1 | -4.60 | -0.04 |
| KJS-96-6b/2 | 1 | -4.19 | 0.50 |
| HP-95-58/1 | 2 | -3.75 | 0.30 |
| HP-95-58/10 | 2 | -6.66 | -2.56 |
| HP-95-58/2 | 2 | -3.85 | -1.75 |
| HP-95-58/3 | 2 | -5.03 | 1.55 |
| HP-95-58/4 | 2 | -5.18 | -0.86 |
| HP-95-58/6 | 2 | -8.38 | -2.01 |
| HP-95-58/7 | 2 | -8.41 | -2.25 |
| HP-95-58/8 | 2 | -6.91 | -1.17 |
| HP-95-58/9 | 2 | -4.03 | -2.40 |
| KJS-96-2/1 | 2 | -2.11 | -0.65 |
