# Supplementary material for: Oldest Evidence of Toolmaking Hominins in a Grassland-Dominated Ecosystem
Source: PLoS One. 2009 Oct 21;4(9):e7199. doi: 10.1371/journal.pone.0007199 (PMC2746317; doi:10.1371/journal.pone.0007199)
Supplement: Table S3 — Vertebrate taxon list from KS-2, Excavation 1. Isotopic dietary classification of Kanjera mammalian fossils follows others (23) using the isotopic data presented in Table S4. Obligate grazers and obligate browsers consume an almost exclusive (>95%) C4 or C3 diet, respectively. Variable grazers and variable browsers consume a predominantly (75–95%) C4 or C3 diet, respectively. Brower-grazer intermediate refers to taxa consuming a mix of C4 and C3 vegetation. (0.04 MB DOC) [file pone.0007199.s003.doc]

|  | **Taxon** | **Predicted Habitat Preference & Diet** | **Isotopic dietary classification** |
| --- | --- | --- | --- |
| Reptilia | Crocodile |  |  |
|  | Chelonia |  |  |
| Aves | Phalacrocoracidae |  |  |
|  | Phasianidae |  |  |
| Mammalia | Hystricidae |  |  |
|  | *Cercopithecus* sp. | Woodland: Fruit&leaves | Browser-grazer intermediate |
|  | *Theropithecus sp.* | Grassland: grass | Variable grazer |
|  | Suidae, indeterminate | Indeterminate | Variable grazer |
|  | *Metridiochoerus modestus* | Woodland/bushland: grass | Variable grazer |
|  | Hippopotamidae | Aquatic: grass | Variable to obligate grazer |
|  | Felidae, leopard-sized |  |  |
|  | Hyaenidae |  |  |
|  | Giraffidae | Woodland: leaves | No data |
|  | Tragelaphini, size 3a | Woodland: leaves | Browser-grazer intermediate |
|  | *Hippotragus sp*. size 3 | Grassland/woodland ecotone: grass | No data |
|  | *Antidorcus* *recki* | Grassland/bushland: mixed feeding | Variable grazer |
|  | Alcelaphini, size 3b | Grassland: grass | Obligate grazer |
|  | Alcelaphini cf. *Parmularius* *altidens* | Grassland: grass | Obligate grazer |
|  | Reduncini, size 1 | Edaphic grassland/woodland: fresh grass | Variable grazer |
|  | Reduncini, size 2 | Edaphic grassland/woodland: fresh grass | Variable grazer |
|  | *Kobus* sp. | Edaphic grassland/woodland: fresh grass | Variable grazer |
|  | *Equus* sp. | Grassland: grass | Variable grazer |
|  | *Eurygnathohippus* sp. | Grassland: grass | Variable grazer |
|  | Rhinocerotidae cf. *Ceratotherium* | Grassland: grass | Obligate grazer |
|  | Elephantidae | Woodland & grassland: mixed feeding | Variable grazer |
|  | *Deinotherium* sp. | Woodland: browse | Variable browser |
