# Supplementary material for: Oldest Evidence of Toolmaking Hominins in a Grassland-Dominated Ecosystem
Source: PLoS One. 2009 Oct 21;4(9):e7199. doi: 10.1371/journal.pone.0007199 (PMC2746317; doi:10.1371/journal.pone.0007199)
Supplement: Table S4 — Stable isotopic composition of fossil eggshell and tooth enamel from Excavation 1. (0.13 MB DOC) [file pone.0007199.s004.doc]

| **Field #** | **Bed** | **Specimen** | **δ ^13^C PDB (Craig corrected)** | **δ^18^O PDB (Craig corrected)** | **Sampled material** |
| --- | --- | --- | --- | --- | --- |
| 5034 | KS-1 | Aves, Struthionidae cf. *Struthio* | -1.841 | 3.368 | shell |
| 2218 | KS-2 | Cercopithecidae | 0.763 | -1.215 | enamel |
| 859 | KS-2 | *Theropithecus* | 0.572 | -0.817 | enamel |
| 10501 | KS-2 | *Cercopithecus* sp. | -1.384 | 0.468 | enamel |
| 3388 | KS-2 | *Equus* sp. | 1.283 | -1.521 | enamel |
| 700 | KS-2 | *Equus* sp. | 1.617 | 0.615 | enamel |
| 160 | KS-2 | *Equus* sp. | 1.670 | 0.812 | enamel |
| 3934 | KS-1 | *Equus* sp. | 1.602 | -0.678 | enamel |
| 3905 | KS-1 | *Eurygnathohippus* sp. | 0.908 | -0.660 | enamel |
| 3664 | KS-1 | *Eurygnathohippus* sp. | 1.171 | -0.942 | enamel |
| 102 | KS-2 | *Eurygnathohippus* sp. | 1.185 | 0.620 | enamel |
| 66 | KS-2 | *Eurygnathohippus* sp. | 1.344 | -0.635 | enamel |
| 2871 | KS-2 | *Eurygnathohippus* sp. | 1.476 | -1.855 | enamel |
| 865 | KS-2 | *Eurygnathohippus* sp. | 1.997 | -1.364 | enamel |
| 5386 | KS-1 | Suidae | -0.932 | -1.016 | enamel |
| 4718 | KS-2 | Suidae | -1.720 | -2.677 | enamel |
| 3775 | KS-2 | Suidae | -0.885 | -0.596 | enamel |
| 14363 | KS-2 | Suidae, cf. *Metridiochoerus* | -0.007 | -0.745 | enamel |
| KJ94-28 | surf KS-1 to 3 | *Metridiochoerus* sp | 1.045 | -1.981 | enamel |
| 5888 | surf KS-1 to 3 | *Metridiochoerus* sp | 1.246 | -0.452 | enamel |
| KJ95-17 | surf KS-1 to 3 | *Metridiochoerus andrewsi* | 1.741 | -1.385 | enamel |
| 5162 | KS-2 | *Metridiochoerus modestus* | 0.549 | -0.997 | enamel |
| 8 | KS-2 | Hippopotamidae | 0.092 | -2.153 | enamel |
| 5004 | KS-2 | Hippopotamidae | 0.120 | -1.174 | enamel |
| 10188 | KS-2 | Hippopotamidae | 0.386 | -2.829 | enamel |
| 8898 | KS-2 | Hippopotamidae | 1.683 | -0.317 | enamel |
| 7211 | KS-2 | Hippopotamidae | 2.041 | 0.238 | enamel |
| 792 | KS-2 | Hippopotamidae | 2.051 | -1.626 | enamel |
| 17990 | KS-2 | Hippopotamidae | 2.661 | -1.178 | enamel |
| 13490 | KS-2 | *Tragelaphini*, size 3a | -2.473 | -0.374 | enamel |
| 3333 | KS-2 | *Tragelaphini*, size 3a | -1.655 | 0.057 | enamel |
| 632 | KS-2 | Alcelaphini cf. *Parmularius altidens* | 1.967 | -0.527 | enamel |
| 755 | KS-2 | Alcelaphini cf. *Parmularius altidens* | 2.063 | -0.485 | enamel |
| 5233 | KS-2 | Alcelaphini cf. *Parmularius altidens* | 2.505 | -0.582 | enamel |
| 5475 | KS-2 | Alcelaphini cf. *Parmularius altidens* | 2.947 | -0.959 | enamel |
| 4300 | KS-2 | *Alcelaphini*, size 3b | 2.899 | -0.607 | enamel |
| 17469 | KS-2 | *Alcelaphini*, size 3b | 3.318 | -1.283 | enamel |
| 21420 | KS-2 | *Antidorcas recki* | -0.419 | 0.417 | enamel |
| 14416 | KS-2 | *Antidorcas recki* | 0.537 | 0.514 | enamel |
| 732 | KS-2 | *Antidorcas recki* | 0.834 | -0.392 | enamel |
| 14655 | KS-2 | *Antidorcas recki* | 0.881 | 0.327 | enamel |
| 4172 | KS-2 | *Antidorcas recki* | 0.916 | -0.335 | enamel |
| 14452 | KS-2 | *Antidorcas recki* | 1.009 | -0.799 | enamel |
| 20515 | KS-2 | *Antidorcas recki* | 1.033 | 1.435 | enamel |
| 11317 | KS-2 | *Antidorcas recki* | 1.256 | 1.290 | enamel |
| 166 | KS-2 | *Antidorcas recki* | 1.231 | 1.595 | enamel |
| 4320 | KS-2 | *Antidorcas recki* | 1.248 | 1.635 | enamel |
| 5165 | KS-2 | *Antidorcas recki* | 1.567 | 1.406 | enamel |
| 15580 | KS-2 | *Antidorcas recki* | 1.946 | 0.704 | enamel |
| 4313 | KS-2 | *Antidorcas recki* | 2.773 | -1.114 | enamel |
| 12522 | KS-2 | *Antidorcas recki* | 0.590 | 0.046 | enamel |
| 21053 | KS-2 | *Antidorcas recki* | 1.934 | 0.272 | enamel |
| 18386 | KS-2 | *Antidorcas recki* | 1.953 | -0.066 | enamel |
| 17737 | KS-2 | *Antidorcas recki* | 2.369 | -0.080 | enamel |
| 12043 | KS-2 | *Reduncini*, size 2 | -0.256 | -1.034 | enamel |
| 286 | KS-2 | *Reduncini*, size 2 | 1.979 | -2.936 | enamel |
| 2168 | KS-2 | *Kobus sp.* | 0.328 | 0.881 | enamel |
| 23316 | KS-2 | *Kobus sp.* | 0.677 | -3.399 | enamel |
| 12045 | KS-2 | *Kobus sp.* | 2.424 | -0.562 | enamel |
| 9 | KS-2 | *Kobus sp.* | 2.578 | -0.794 | enamel |
| 3654 | KS-2 | *Bovini*, size 5 | 1.760 | -0.743 | enamel |
| 8512 | KS-3 | Elephantidae | 1.973 | -2.481 | enamel |
| 5873 | surf KS-1 to 3 | Elephantidae | 1.611 | -2.485 | enamel |
| KJ95a | surf KS-1 to 3 | *Deinotherium* sp. | -10.616 | -2.370 | enamel |
| KJ95b | surf KS-1 to 3 | *Deinotherium* sp. | -7.556 | -2.292 | enamel |
| 6291 | surf KS-1 to 3 | Rhinocerotidae, cf *Ceratotherium* | 2.224 | -0.580 | enamel |
| 2066 | surf KS-1 to 3 | Hyaenidae cf. *Crocuta* | 0.801 | -1.621 | enamel |
